# Association Between Kyphosis and Sleep Disturbance in Community-Dwelling Older Adults: The Locomotive Syndrome and Health Outcome in Aizu Cohort Study

**DOI:** 10.7759/cureus.76722

**Published:** 2025-01-01

**Authors:** Takuya Nikaido, Koji Otani, Miho Sekiguchi, Shingo Fukuma, Tsukasa Kamitani, Kazuyuki Watanabe, Kinshi Kato, Hiroshi Kobayashi, Masataka Nakamura, Ryoji Tominaga, Shoji Yabuki, Shin-ichi Konno, Yoshihiro Matsumoto

**Affiliations:** 1 Department of Orthopaedic Surgery, Fukushima Medical University School of Medicine, Fukushima, JPN; 2 Department of Epidemiology Infectious Disease Control and Prevention, Hiroshima University Graduate School of Biomedical and Health Sciences, Hiroshima, JPN; 3 Department of Human Health Sciences, Kyoto University Graduate School of Medicine, Kyoto, JPN; 4 Department of Education for Clinical Research, Kyoto University Hospital, Kyoto, JPN; 5 Institute for Health Outcomes and Process Evaluation Research, iHope International, Kyoto, JPN; 6 Department of Research for Spine and Spinal Surgery, Fukushima Medical University, Fukushima, JPN; 7 Department of Orthopaedic Surgery, Fukushima Medical University, Fukushima, JPN; 8 Department of Orthopaedics, Iwai Orthopaedic Medical Hospital, Tokyo, JPN

**Keywords:** adult spinal deformity, kyphosis, lohas, older adults, sleep disturbance

## Abstract

Purpose: Worsening sagittal alignment of the spine, particularly kyphosis, may cause difficulty in assuming a supine position, restricting sleeping posture and movement and potentially leading to sleep disturbances. However, no studies have explored the relationship between sagittal spinal alignment and sleep disturbance. This study aimed to clarify the relationship between sagittal spinal alignment and sleep disturbance.

Methods: Data were drawn from the Locomotive Syndrome and Health Outcome in Aizu Cohort Study (LOHAS) in 2010. Sleep disturbance was assessed using a self-administered questionnaire on average sleep duration over the past month, with patients classified as having sleep disturbance if they slept for fewer than six hours per day. The sagittal vertical axis (SVA) was measured on standing whole-spine radiographs and classified into three groups based on the Scoliosis Research Society Schwab classification: non-kyphosis: <40 mm; moderate kyphosis: 40-95 mm; and severe kyphosis: >95 mm. Age, sex, drinking habits, depressive symptoms, overactive bladder symptoms, and napping habits were assessed as confounding factors. The association between SVA and sleep disturbance was evaluated using adjusted odds ratios and 95% confidence intervals (CIs).

Results: The percentage of sleep disturbance among the 772 subjects in the analysis was 8.9% for non-kyphosis, 9.1% for moderate kyphosis, and 20.0% for severe kyphosis. Using non-kyphosis as the reference, adjusted odds ratios (ORs; 95% confidence intervals (95% CIs)) were 1.16 (0.65-2.05) for moderate kyphosis and 2.86 (1.13-7.26) for severe kyphosis.

Conclusion: Kyphosis in community-dwelling adults was found to be associated with sleep disturbance. Therefore, it is necessary to focus on the parasomnias of sleep disturbance in patients presenting with spinal kyphosis.

## Introduction

Spinal kyphosis is the most common sagittal malalignment in older individuals, and its prevalence increases with advancing age. Although the exact prevalence of kyphosis in older individuals is unknown, it has been reported to range between 20% and 40% [1-3]. Kyphosis often results in pain in the back and lower extremities, along with musculoskeletal problems such as mobility deficits, impaired gait, and a high incidence of falls [4-8]. Hyperkyphosis, defined as a kyphotic angle greater than 40° [3,9], is associated with an increased risk of falls [8]. Falls in older adults can lead to fractures, potentially resulting in a patient becoming bedridden [10,11]. That is, kyphosis triggers a negative chain of events, affecting locomotion and increasing the risk of premature mortality [12]. Furthermore, kyphosis is associated with several medical complications, including pulmonary dysfunction and gastroesophageal reflux disease [13-18]. Mental health issues, such as diminished self-image, reduced motivation, and social withdrawal, are also associated with kyphosis in elderly individuals [18-20].

Aging is also associated with changes in sleep quality and duration, with sleep problems becoming more prevalent after the age of 65 years [21-23]. Approximately 50% of elderly individuals report difficulty sleeping [24,25]. In addition, the prevalence and incidence of depression are increasing in older individuals, affecting women twice as often as men. Minor depression has a prevalence of 4%-13% [26,27]. Changes in sleep neurophysiology are often observed in patients with depression, and impaired sleep is often the chief complaint [28-30]. In patients with chronic spinal pain, the prevalence of poor sleep is estimated to be as high as 64% [31,32]. Poor sleep and spinal pain are both important factors contributing to psychological distress [33]. Old age, spinal pain, depression, and sleep disturbance are thus closely interrelated.

Spinal sagittal malalignment, including kyphosis, is expected to cause sleep disturbances by restricting sleeping posture and limiting rolling motion. However, few studies have explored the relationship between kyphosis and sleep disturbances, and this connection has received little attention in research [12,34]. A cross-sectional study by Wankie et al. reported that older women had worse sleep quality than men [12]. In contrast, a cohort study by Kaufmann found no association between kyphosis and sleep quality in elderly men [34]. Notably, both studies relied on qualitative assessments of kyphosis, and the relationship between radiographic kyphosis and sleep disturbances remains poorly understood. Furthermore, the relationship between the degree of radiological kyphosis and sleep disturbances remains unclear.

This study aimed to clarify the relationship between radiological kyphosis and sleep disturbances using data from the Locomotive Syndrome and Health Outcome in Aizu Cohort Study (LOHAS) [35].

## Materials and methods

Study population

The LOHAS is a population-based cohort designed to evaluate the risks of cardiovascular disease, quality of life, medical costs, falls, and mortality related to locomotive dysfunction [35]. The study protocol was approved by the Research Ethics Committee of Fukushima Medical University School of Medicine, Fukushima, Japan (approval no. 673), and all participants provided written informed consent. The LOHAS cohort consists of residents from two communities (Tadami and Minamiaizu towns in Fukushima Prefecture, Japan) aged over 40 years, who underwent regular annual health check-ups conducted by the local government. This study conducted a cross-sectional analysis of the LOHAS data. Eligibility criteria included participants aged >40 years who participated in annual health check-ups and underwent radiographic spinal assessment in 2010. Participants with a history of spinal surgery were excluded. Details of the LOHAS design have been previously published [35].

Spinal kyphosis measurement

Kyphosis was assessed by standing whole-spine radiography. The sagittal vertical axis (SVA) was identified as an indicator of sagittal spinal deformity and was measured by two investigators (RT and YK) as the deviation of the C7 plumb line (originating at the middle of the C7 vertebral body) from the posterior superior endplate of S1 using Surgimap software (Nemaris, Inc., New York, NY, USA) [36]. Intra- and inter-observer reliability for the SVA measurement were 0.984 and 0.998, respectively. For analysis, the first half of the measurements were taken by investigator RT, and the second half of the measurements were taken by investigator YK. The SVA measurements were categorized into three groups according to the sagittal alignment criteria of the Scoliosis Research Society Schwab Adult Spinal Deformity Classification (non-kyphosis: <40 mm; moderate kyphosis: 40-95 mm; severe kyphosis: >95 mm) [37].

Outcome: sleep disturbance

Sleep disturbance was assessed based on the average number of hours of sleep per day over the past month, as reported in a self-administered questionnaire. The American Academy of Sleep Medicine and Sleep Research Society recommends seven or more hours per night for optimal health [38]. However, a global comparison of average sleep hours across 33 countries (Organization for Economic Cooperation and Development (OECD) database) indicated that Japan has the shortest average sleep duration, more than one hour less than the global average [39]. Based on the Japanese Ministry of Health, Labour, and Welfare’s recommendation of six to eight hours of sleep for good health, participants reporting less than six hours of sleep per night were considered to have sleep disturbance.

Measurement of potential confounding variables

Potential confounding variables influencing sleep disturbance were identified and included sociodemographic characteristics, such as age, sex, drinking habits, depressive symptoms, overactive bladder symptoms, and napping habits, all of which were assessed using a self-reported questionnaire.

Drinking habits

Alcohol intake can significantly impact sleep duration and quality. Higher alcohol consumption is associated with increased sleep duration and wake-after-sleep onset (WASO) [40]. Clinical assessment of sleep should include evaluation of alcohol use, as it negatively affects sleep quality and increases WASO and sleep fragmentation [41]. Drinking habits were assessed using the following categories of standard alcohol consumption: <20 g, 20-40 g, 40-60 g, and >60 g.

Depressive symptoms

Sleep disorders and depression are strongly interrelated in epidemiology, clinical presentation, and neurobiology, especially in elderly individuals [42]. Epidemiological studies have indicated that depression is a major risk factor for insomnia [43]. Depressive symptoms were assessed using the 10-item version of the Center for Epidemiological Studies Depression Screening Index (CES-D), which quantifies the frequency and severity of depressive symptoms. A score of ≥10 is considered indicative of depressive mood [44].

Overactive bladder symptoms

Frequent nocturia, often caused by an overactive bladder, contributes to sleep disturbances. Overactive bladder symptoms were assessed using the Overactive Bladder Symptom Score, a four-question questionnaire. A score of ≥2 on Question 3 and a total score of ≥3 on Question 4 are required for the diagnosis and severity of overactive bladder [45].

Napping habits

Research has revealed complex relationships between napping and nighttime sleep. Frequent napping in adolescents has been linked to shorter nocturnal sleep duration and poorer sleep efficiency [46]. In elderly individuals, increased napping is associated with greater sleep fragmentation [47]. Napping habits were assessed via a self-report questionnaire.

Statistical analysis

Participants with no missing data were included in the statistical analyses. To estimate the risk of sleep disturbance, a primary analysis using multiple regression models was performed to calculate odds ratios (ORs) and 95% confidence intervals (95% CIs). The model was adjusted for clinically relevant confounders, including age, sex, drinking habits, depressive symptoms, overactive bladder symptoms, and napping habits. Statistical significance was set at P <0.05. All analyses were conducted using Stata Statistical Software, Release 13 (StataCorp LLC, College Station, TX, USA).

## Results

**Figure 1:**
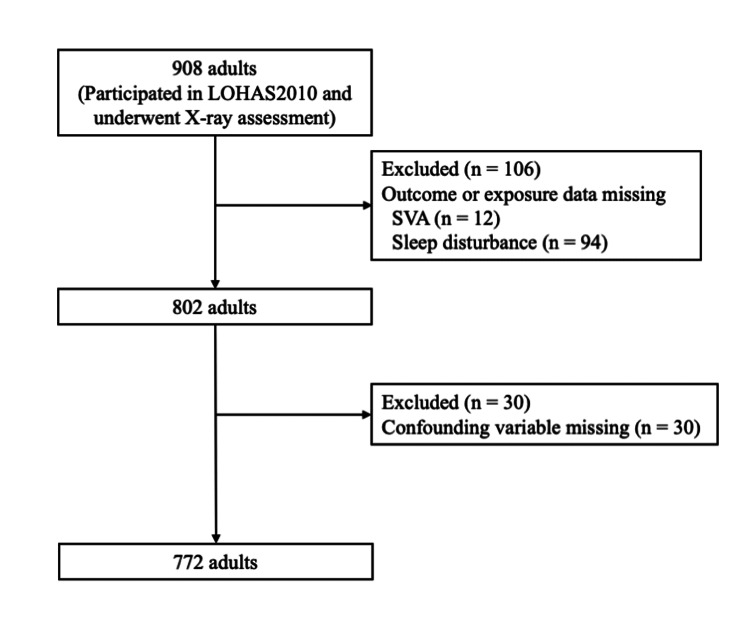
Flowchart of the study population Of the 908 study participants in LOHAS 2010 who underwent X-ray assessment, 772 (85.0%) were included for statistical analysis in this present study. LOHAS: Locomotive Syndrome and Health Outcomes in Aizu Cohort Study; SVA: sagittal vertical axis

Of the 908 participants in the health checkup and X-ray assessment in LOHAS 2010, 802 (88.3%) had both exposure and outcome variables. After excluding 30 participants with at least one missing confounding variable, the remaining 772 (85.0%) were included in the analysis (Figure 1).

The mean age of the participants was 69.7 ± 9.0 years, with 61.7% being female. Table 1 summarizes the baseline characteristics categorized according to the degree of kyphosis, as measured using SVA.

**Table 1:** Characteristics of the study participants based on the three categories of sagittal vertical axis (SVA)

Sagittal balance	Non-kyphosis	Moderate kyphosis	Severe kyphosis	Total
	SVA <40mm	SVA 40-95mm	SVA >95mm	n=772
	n=516, 66.8%	n=221, 28.6%	n=35, 4.5%	
Age, mean [SD]	66.6 (9.3)	70.2 (7.8)	72.4 (7.6)	67.9 (9.0)
Female, n (%)	322 (62.4)	133 (60.2)	21 (60.0)	476 (61.7)
Drinking habits (standard drink), n (%)				
<20g	119 (23.1)	53 (24.0)	6 (17.1)	178 (23.1)
20g≤, <40g	97 (18.8)	37 (16.7)	3 ( 8.6)	137 (17.7)
40g≤, <60g	134 (26.0)	55 (24.9)	8 (22.9)	197 (25.5)
60g≤	166 (32.2)	76 (34.4)	18 (51.4)	260 (33.7)
Depressive symptoms, n (%)	91 (17.6)	38 (17.2)	9 (25.7)	138 (17.9)
Overactive bladder symptoms, n (%)	51 ( 9.9)	30 (13.6)	4 (11.4)	85 (11.0)
Napping habits, n (%)	203 (39.3)	82 (37.1)	17 (48.6)	302 (39.1)

The prevalence of non-kyphosis, moderate kyphosis, and severe kyphosis was 66.8% (n=516), 28.6% (n=221), and 4.5% (n=35), respectively. All three groups had a higher prevalence of drinking habits, with the highest proportion consuming >60 g of standard alcohol. The prevalence of depressive symptoms, as per the CES-D criteria, was 17.6% for non-kyphosis, 17.2% for moderate kyphosis, and 25.7% for severe kyphosis. The prevalence of overactive bladder symptoms causing nocturia that affected sleep was 9.9% in the non-kyphosis group, 13.6% in the moderate kyphosis group, and 11.4% in the severe kyphosis group. The proportion of participants with a napping habit that might affect nighttime sleep duration was 39.3% for the non-kyphosis group, 37.1% for the moderate kyphosis group, and 48.6% for the severe kyphosis group.

The prevalence of sleep disturbance across the SVA categories was 8.9% for non-kyphosis, 9.1% for moderate kyphosis, and 20.0% for severe kyphosis (Table 2).

**Table 2:** Prevalence of sleep disturbance among the three categories of sagittal vertical axis (SVA)

Sagittal balance	Non kyphosis	Moderate kyphosis	Severe kyphosis
	SVA <40mm	SVA 40-95mm	SVA >95mm
Average sleeping hours per day, less than six hours (%)	8.9	9.1	20.0

In the multivariable logistic regression model adjusted for age, sex, drinking habits, depressive symptoms, overactive bladder symptoms, and napping habits, the adjusted OR for sleep disturbance was 1.16 (95% CI 0.65-2.05) for moderate kyphosis and 2.86 (95% CI 1.13-7.26) for severe kyphosis, relative to the non-kyphosis group (Table 3).

**Table 3:** Multivariable logistic regression model with adjustment for age, gender, drinking habits, depressive symptoms, overactive bladder symptoms, and napping habits SVA: sagittal vertical axis; ORs: odds ratios; CI: confidence interval

Sagittal balance	Non-kyphosis	Moderate kyphosis	Severe kyphosis
	SVA <40mm	SVA 40-95mm	SVA >95mm
Adjusted ORs	Ref.	1.16	2.86
(95% CIs)	-	(0.65-2.05)	(1.13-7.26)

## Discussion

Worsening sagittal spinal alignment, particularly kyphosis, can make it difficult to lie supine, restrict sleeping posture and turning movements, and is expected to contribute to sleep disturbances. Sleep disturbance, in turn, is a significant risk factor for falls and fractures, which are particularly concerning issues for elderly individuals. Poor sleep quality in older adults is associated with numerous health issues and a reduced quality of life, including cognitive impairment, an increased risk of falls, and higher mortality rates [48]. In addition, sleep disturbances in elderly individuals are associated with declined functional status, reduced social functioning, and a significantly impaired quality of life [49]. However, only a few studies have examined the relationship between kyphosis and sleep disturbance. As society ages, understanding this relationship between kyphosis and sleep disturbance is of great social importance.

In this study of community-dwelling adults, kyphosis was associated with sleep disturbance over the past month, even after adjusting for age, sex, drinking habits, depressive symptoms, overactive bladder symptoms, and napping habits. Adjusted OR for these factors was 1.16 in the group with SVA 40-95 mm and 2.86 in the group with SVA >95 mm compared to the group without kyphosis. These findings suggest that kyphosis is an independent risk factor for sleep disturbance, particularly as the severity of kyphosis increases. A major strength of this study is the quantitative radiological assessment of kyphosis and the analysis of the association between the degree of kyphosis, which provides a more objective measure compared to previous studies, and the adjustment of confounding factors affecting sleep disturbance.

In a prior study, we found that lumbar spinal stenosis was an independent risk factor for sleep disorders [50], highlighting the comorbidity between sleep disorders and spinal diseases. Wankie et al. reported that older women with kyphosis had poorer self-reported sleep quality, although no such association was found in men [12]. Kaufmann et al. also observed that poor posture and worsening kyphosis in older men with severe kyphosis were not associated with poor sleep quality, based on either self-reports or objective measures [35]. However, these studies only qualitatively measured kyphosis and did not perform a highly objective radiological assessment. This study, by contrast, is the first to investigate the association between the degree of kyphosis and sleep disturbance using an objective radiological measure of sagittal spinal alignment. In addition, multivariate analyses adjusted for age, sex, drinking habits, depression symptoms, overactive bladder symptoms, and napping habits were conducted, ensuring the high reliability of the results.

The findings of this study suggest that detailed evaluations should be conducted with patients with kyphosis, specifically assessing the presence of sleep disturbances, and that aggressive interventions should be implemented in cases of sleep disturbances. Treatment options for sleep disturbances include conservative methods such as pharmacotherapy and cognitive behavioral therapy [51,52], as well as surgical treatment for kyphosis. However, surgical correction is often associated with a high frequency of serious complications, so the indications for surgery should be carefully considered [53,54]. In addition to preventing impairments in activities of daily living and quality of life due to kyphosis, approaches to prevent the worsening of kyphosis, such as strengthening the trunk muscles [55,56] and early intervention for vertebral fractures [57], are important from the perspective of managing sleep disturbances.

This study has some limitations. First, the radiological evaluation was performed using standing X-rays, which did not assess postural alignment or spinal mobility during sleep. Hasegawa et al. reported that radiographic spinal sagittal parameters from the lumbar spine to the pelvis in the standing position were significantly worse in patients with adult spinal deformity than in the supine position, although the degree of thoracic kyphosis was not significantly altered [58]. Therefore, it is unclear whether spinal alignment in the upright position in normal subjects accurately reflects the posture during sleep. Second, the short sleep duration used as the outcome in this study may not fully reflect sleep disorders. Sleep disorders include not only short sleep duration but also declines in sleep quality and symptoms like hypersomnia and parasomnias, which are also important indicators of sleep disorders that were not assessed in this study. Furthermore, the average sleep time per day was self-reported and may not accurately reflect this. Third, some of the confounding factors, such as napping habits, may be intermediate factors or consequences of short sleep duration, rather than independent confounders.

## Conclusions

Kyphosis is an independent risk factor for sleep disturbances, with the severity of kyphosis increasing the likelihood of sleep disturbances. Therefore, it is crucial to closely monitor and address sleep disturbances in patients with spinal kyphosis, particularly those with more severe forms. Early identification and intervention for sleep disorders in these patients can help improve their overall health and quality of life.
